# Refractory Intrahepatic Cholestasis of Pregnancy in Twin Gestation Managed With Ursodeoxycholic Acid and Adjunctive Cholestyramine: A Case Report

**DOI:** 10.7759/cureus.101309

**Published:** 2026-01-11

**Authors:** Samah A ElSalem, Sadia Mehmood, Afif Ahmed, Muna Al-Saadi

**Affiliations:** 1 Pharmacy, Hamad Bin Khalifa Medical City (HBKMC), Doha, QAT; 2 Obstetrics and Gynecology, Hamad Medical Corporation, Doha, QAT

**Keywords:** bile acids, cholestasis, cholestyramine resin, intrahepatic cholestasis, multiple pregnancy, refractory intrahepatic cholestasis, ursodeoxycholic acid

## Abstract

Intrahepatic cholestasis of pregnancy (ICP) is a liver disorder which is characterized by pruritus and elevated bile acids during pregnancy and is further coupled with higher maternal and fetal risks. We report a rare case of early-onset, refractory ICP with elevated liver enzymes in a 25-year-old primigravida with a monochorionic diamniotic twin gestation complicated by selective fetal growth restriction (sFGR). At 24 weeks of gestation, the patient presented with severe generalized pruritus, mainly on the palms and soles, impairing her quality of life. The patient with insignificant medical history of liver diseases, but has a significant family history of ICP. On examination, she was afebrile, hemodynamically stable, and without jaundice or rash. Laboratory findings revealed moderately raised bile acids (40.98 µmol/L) fulfilling criteria for moderate ICP but coupled with elevated liver enzymes. Viral hepatitis serologies, autoimmune profile, and abdominal ultrasound were all unremarkable. Treatment with ursodeoxycholic acid (UDCA) 500 mg twice daily was initiated. Given worsening biochemical (doubling of bile acids) and clinical findings, gastroenterologists were consulted after a week of treatment initiation, and their recommendation was to add cholestyramine 4 g once daily as an adjunctive. Afterwards, with persistently elevated bile acids, the UDCA dose increased to 500 mg three times daily, and cholestyramine was increased to 4 g twice daily. Phytonadione (vitamin K) 10 mg orally daily was added to reduce the risk of bleeding due to reduced absorption of fat-soluble vitamins caused by cholestyramine. At 32 weeks of gestation, she developed vomiting, coffee-ground emesis, vaginal spotting, and abdominal pain, progressing to preterm labor with non-reassuring fetal heart tracings. An emergency category 1 cesarean section was performed, delivering two viable female infants (1600 g and 1160 g), both admitted to the neonatal intensive care unit (NICU). Postnatally, the maternal condition was stable, and biochemical parameters normalized rapidly within 72 hours postpartum. Pruritus resolved completely, confirming the diagnosis of ICP.

## Introduction

Intrahepatic cholestasis of pregnancy (ICP) is the most common pregnancy-specific liver disorder, characterized by pruritus, elevated serum bile acids, and abnormal liver function tests [1]. This usually develops in the second or third trimester and resolves spontaneously after delivery [1,2]. Global prevalence varies widely, ranging between 0.2% and 2% [1,2]. Although the ICP prognosis on the mother is generally favorable, ICP is associated with increased risk of adverse perinatal outcomes, including spontaneous preterm birth, meconium-stained amniotic fluid, and intrauterine fetal demise (IUFD), mainly when maternal serum bile acids reach above 100 µmol/L [3].

The pathophysiology of ICP is multifactorial, involving genetic mutations in bile acid transporters, hormonal changes during pregnancy (elevated estrogen and progesterone metabolites), and environmental factors that impair bile acid excretion [4,5]. The first-line management of ICP is ursodeoxycholic acid (UDCA), which improves pruritus and biochemical parameters, though its effect on reducing IUFD remains controversial [6].

According to the Royal College of Obstetricians and Gynaecologists (RCOG) Guideline (2022), ICP severity is classified into different severities by bile acid levels: mild ICP (19-39 µmol/L), moderate ICP (40-99 µmol/L), and severe ICP (≥100 µmol/L) [7]. The RCOG guideline stresses the need for close monitoring of women with moderate-to-severe ICP, with consideration of planned delivery at 35-37 weeks depending on bile acid levels, obstetric history, and presence of refractory symptoms [7]. For women with severe pruritus not adequately relieved by UDCA, RCOG suggests an individualized approach, which may include an increased UDCA dose, addition of symptomatic treatments (such as emollients and/ or antihistamines), and the addition of rifampicin as an adjunctive treatment in refractory cases [7-9].

Cholestyramine, a bile acid-binding resin, has been used as a second-line or adjunctive medication in refractory cases. It works by interrupting enterohepatic circulation and enhancing fecal excretion of bile acids [10]. Despite being less effective than UDCA in reducing pruritus and having minimal impact on bile acid levels, it can provide partial symptomatic relief when used as an adjunctive with UDCA in women with severe or persistent symptoms [10-12]. The use of cholestyramine may be limited by gastrointestinal side effects and interference with the absorption of fat-soluble vitamins, mainly vitamin K, requiring monitoring and supplementation if prolonged therapy is required [10,11].

Early recognition and aggressive management of refractory ICP are important to reduce maternal discomfort, improve biochemical control, and minimize the risk of IUFD. Here, we present a case of antenatal severe ICP with pruritus unrelieved by UDCA, requiring adjunctive cholestyramine, which completely resolved postpartum.

## Case presentation

A 25-year-old primigravida (G1P0) with a monochorionic diamniotic twin pregnancy, at 24 + 2 weeks of gestation, presented to the antenatal clinic with a seven-day history of severe generalized pruritus, mainly on the palms and soles, significantly impairing sleep and quality of life. She had no past medical history of liver or biliary disease. Her obstetric history was insignificant. Her family history was significant for ICP in a first-degree relative (sister). On examination, she was afebrile, hemodynamically stable, with no jaundice or skin rash.

Initial laboratory work-up showed elevated liver transaminases (ALT: 170.4 U/L; AST: 68 U/L) and mildly elevated serum bile acids (40.98 µmol/L), meeting the criteria for moderate ICP. Other investigations, including hepatitis viral serologies, autoimmune profile, and abdominal ultrasound, were unremarkable. Tables 1-2 show the profiles of bile acids, alanine aminotransferase (ALT), and aspartate aminotransferase (AST) throughout pregnancy and postpartum, respectively.

**Table 1:** Bile acid profile throughout pregnancy and postpartum *ICP: intrahepatic cholestasis of pregnancy

Gestational age/postpartum time	Patient’s bile acids (µmol/L)	Bile acids (reference range) (µmol/L)
*ICP diagnosis (24 weeks)	40.98	2-10
25 weeks	82.48	2-10
27 weeks	72.08	2-10
27 weeks	115.32	2-10
27 weeks	73.48	2-10
28 weeks	78.65	2-10
28 weeks	68.51	2-10
28 weeks	70.82	2-10
28 weeks	100.34	2-10
28 weeks	112.66	2-10
28 weeks	94.43	2-10
29 weeks	78.51	2-10
29 weeks	46.75	2-10
29 weeks	76.25	2-10
29 weeks	67.1	2-10
29 weeks	73.41	2-10
30 weeks	39.33	2-10
30 weeks	98.89	2-10
30 weeks	39.98	2-10
30 weeks	125.75	2-10
30 weeks	74.49	2-10
31 weeks	58.3	2-10
31 weeks	86.72	2-10
31 weeks	63.52	2-10
31 weeks	51.39	2-10
31 weeks	84.8	2-10
31 weeks	73.2	2-10
Day of delivery (32 weeks)	81.05	2-10
Day 0 postpartum	27.47	2-10
Day 1 postpartum	16.68	2-10
Day 3 postpartum	9.76	2-10

**Table 2:** ALT and AST levels throughout pregnancy and postpartum *ICP: intrahepatic cholestasis of pregnancy; **ALT: alanine aminotransferase; ***AST: aspartate aminotransferase

Gestational age/postpartum time	Patient’s **ALT (U/L)	**ALT reference range (U/L)	Patient’s ***AST (U/L)	***AST reference range (U/L)
*ICP diagnosis (24 weeks)	170.4	0-33	68	0-32
25 weeks	298	0-33	295	0-32
25 weeks	556	0-33	312	0-32
27 weeks	558	0-33	258	0-32
27 weeks	454	0-33	241	0-32
27 weeks	418	0-33	504	0-32
28 weeks	654	0-33	392	0-32
28 weeks	578	0-33	281	0-32
28 weeks	452	0-33	214	0-32
28 weeks	391	0-33	190	0-32
28 weeks	337	0-33	183	0-32
29 weeks	326	0-33	194	0-32
29 weeks	322	0-33	205	0-32
29 weeks	304	0-33	213	0-32
29 weeks	297	0-33	234	0-32
29 weeks	326	0-33	216	0-32
29 weeks	297	0-33	230	0-32
30 weeks	296	0-33	192	0-32
30 weeks	247	0-33	102	0-32
30 weeks	153	0-33	103	0-32
30 weeks	145	0-33	-	0-32
31 weeks	127	0-33	103	0-32
31 weeks	142	0-33	114	0-32
31 weeks	139	0-33	111	0-32
31 weeks	120	0-33	96	0-32
31 weeks	131	0-33	110	0-32
31 weeks	121	0-33	98	0-32
Day of delivery (32 weeks)	118	0-33	93	0-32
Day 0 postpartum	132	0-33	110	0-32
Day 1 postpartum	95	0-33	90	0-32
Day 2 postpartum	59	0-33	44	0-32
Day 3 postpartum	49	0-33	31	0-32
Day 5 postpartum	36	0-33	18	0-32

She was started on UDCA 500 mg twice daily (17 mg/kg/day), along with topical emollients and oral antihistamines for symptomatic relief. After one week, her pruritus remained severe with no improvement, and her bile acid levels nearly doubled to 82.48 µmol/L (Table 1), coupled with worsening liver function tests, despite full adherence to UDCA therapy. At 26+ weeks of gestation, the patient was referred to gastroenterology, and following multidisciplinary discussion, the gastroenterology team recommended initiating cholestyramine 4 g orally once daily as an adjunct for refractory symptoms. Following initiation of cholestyramine 4 g once daily, the patient reported improvement in pruritus, which became localized to the soles.

Later on, the patient was admitted to the obstetrics and gynecology ward for further management and close monitoring. A detailed ultrasound by the Fetal Medicine Unit (FMU) confirmed a dichorionic twin pregnancy. Fetus A, in cephalic presentation on the maternal left, was small for gestational age with an estimated growth at the 1st percentile, reduced amniotic fluid, and normal Doppler studies, with no evidence of fetal anemia. Fetus B, in breech presentation on the maternal right, was appropriate for gestational age with normal amniotic fluid and Doppler findings. The impression was selective fetal growth restriction (sFGR) in Twin A, with no sonographic evidence of twin-to-twin transfusion syndrome (TTTS). These findings, in the context of ICP and a multifetal gestation, highlighted the complexity of her case and the heightened risk for adverse perinatal outcomes, necessitating intensive surveillance and multidisciplinary involvement. The case was discussed with the obstetrics team, and at 27+ weeks of gestation, a course of betamethasone (12 mg intramuscularly every 24 hours for two doses) was administered to promote fetal lung maturation due to risk of preterm delivery.

At 28+ weeks of gestation, in the context of worsening bile acid levels and liver function tests, the UDCA dose was increased to 500 mg three times daily (25 mg/kg/day) while the patient continued cholestyramine 4 g orally once daily as adjunct therapy. Following multidisciplinary discussion with gastroenterology and senior obstetrics consultants, the cholestyramine dose was recommended to be increased to 4 g orally twice daily, and vitamin K 10 mg orally daily was added to prevent the risks of inadequate vitamin K absorption. The obstetricians highlighted that maternal serum bile acids above 100 µmol/L are associated with increased fetal risk, and early delivery should be considered if levels remain high. The obstetrics team advised continued inpatient management due to persistently elevated bile acids and the presence of a monochorionic diamniotic twin pregnancy with selective intrauterine growth restriction (sIUGR) of twin A. The patient was to remain inpatient with daily monitoring of maternal serum bile acids. Delivery was planned between 32 and 34 weeks, unless maternal bile acids reached 200 µmol/L or higher, in which case immediate delivery would be indicated.

At 32 weeks of gestation, the patient developed 2-3 episodes of coffee-ground emesis and heartburn, along with a single episode of dark-colored, constipated stool without frank blood. She also reported poor appetite and reduced oral intake over the preceding days. Subsequently, she experienced vaginal spotting and the onset of epigastric and lower abdominal pain. On assessment, she appeared unwell and in pain, with clinical signs suggestive of labor. Vaginal examination revealed cervical dilatation of 3 cm with 90% effacement and intact membranes. Fetal assessment confirmed positive heart activity in both twins, though cardiotocography (CTG) of one twin showed reduced variability and variable decelerations, raising concern for fetal compromise. In view of her acute presentation and non-reassuring fetal status, an emergency category 1 lower-segment cesarean section (LSCS) was performed.

Two female infants were delivered via emergency cesarean section at 32 weeks of gestation. The first twin, presenting breech, was delivered breech with a birth weight of 1600 g. Apgar scores were 8 and 9 at one and five minutes, respectively. The second twin, also presenting breech, was delivered breech with a birth weight of 1160 g. Apgar scores were 2 and 9 at one and five minutes, respectively. Both neonates required admission to the neonatal intensive care unit (NICU) for preterm management.

Throughout her antenatal treatment and the postpartum period, the patient’s coagulation profile remained within normal limits, including prothrombin time (PT), activated partial thromboplastin time (aPTT), and international normalized ratio (INR), with no evidence of prolongation or coagulopathy at any point.

The maternal postoperative course was uneventful except that the patient continued to experience constipation and symptoms of acidity. Laboratory evaluation revealed a significant postpartum drop in hemoglobin from 10.1 g/dL on day 1 post-LSCS to 7.4 g/dL on day 2, indicating acute blood loss anemia, which was managed accordingly. Serum bile acid levels began to decline within 24 hours of delivery and normalized within 72 hours (Table1). Liver function tests improved as well, with AST normalizing within 72 hours; however, the last available ALT value remained mildly elevated at 36 U/L (normal 0-33) on day 4 postpartum (Table 2). Pruritus completely resolved following delivery. This clinical course, characterized by rapid postpartum biochemical and symptomatic resolution, confirmed the diagnosis of ICP.

## Discussion

ICP is a liver disorder unique to pregnancy, usually present during the second or third trimester with pruritus, mainly affecting the palms and soles, and biochemical abnormalities, including elevated serum bile acids and liver transaminases [13,14]. ICP is associated with increased maternal and perinatal risks, especially in multiple gestations, where the incidence and severity may be higher [15]. Our case highlights several important clinical considerations: early-onset, refractory ICP in a monochorionic diamniotic twin pregnancy with sIUGR, management challenges, and the role of adjunctive therapy.

This case report describes a pregnant patient at 24 + 2 weeks of gestation with severe pruritus and moderate biochemical abnormalities, meeting the criteria for ICP. She had a positive family history, which is considered a risk factor [16]. Initial management with UDCA failed to provide symptomatic or biochemical improvement, with bile acids nearly doubling after one week despite full patient adherence to medication. This scenario represents refractory ICP, defined by persistent pruritus and worsening biochemical parameters despite optimum therapy [17].

Given the persistent symptoms and elevated bile acids, the patient was referred to gastroenterology and a multidisciplinary team, who recommended adjunctive cholestyramine therapy. Cholestyramine, a bile acid-binding resin, has been reported in limited studies as an effective adjunctive therapy for symptomatic relief in patients who are on UDCA with persistent symptoms [18,19]. However, despite symptomatic relief, evidence for positive cholestyramine effect on fetal outcomes is limited [18,19]. In this case report, cholestyramine resulted in partial symptomatic improvement and was safely combined with UDCA and vitamin K supplementation to prevent fat-soluble vitamin K deficiency [14].

Cholestyramine use has been associated with hemorrhagic complications in patients, including epistaxis, bruising, genitourinary bleeding, gastrointestinal bleeding, and periarticular bleeding [20]. These events are often reversible upon discontinuation, although recurrence has been reported with reinitiation of cholestyramine [18,20]. The mechanisms of cholestyramine-associated hemorrhage include malabsorption of fat-soluble vitamins, mainly vitamin K, leading to hypoprothrombinemia, and potential mucosal injury due to crystal formation from the ion exchange resin [20]. The onset of bleeding can range from days to years of cholestyramine initiation [18,20]. In this case report, oral vitamin K 10 mg daily was initiated while cholestyramine reduced the risk of bleeding complications, especially when the dosing of cholestyramine increased to twice daily. However, despite the addition of vitamin K, our patient still developed gastrointestinal-related bleeding complications, e.g., 2-3 episodes of coffee-ground emesis and heartburn.

Postpartum, maternal bile acids and liver function tests rapidly normalized within 72 hours, and pruritus resolved completely, which is consistent with the typical postpartum resolution of ICP [13].

This case emphasizes several key points, including early onset, severe ICP, particularly in twin pregnancies, may be refractory to UDCA and require adjunctive therapies such as cholestyramine; also, clinicians should be aware of potential hemorrhagic complications associated with cholestyramine, particularly in the setting of escalating doses or chronic use, and consider vitamin K supplementation. Moreover, this case emphasizes the need for close maternal and fetal monitoring, multidisciplinary management, and timely delivery, which are critical to optimize outcomes.

Finally, this case highlights the challenges of managing refractory ICP in a twin gestation with sIUGR, which supports the potential role of cholestyramine as adjunctive therapy and emphasizes the importance of individualized, multidisciplinary care to achieve favorable maternal and neonatal outcomes while reducing therapy-related risks.

## Conclusions

This case illustrates the challenges of managing early-onset, refractory ICP in a monochorionic diamniotic twin gestation complicated by SIUGR. Despite standard therapy with UDCA, the patient’s symptoms and biochemical markers worsened, which required adjunctive cholestyramine therapy. Careful dose titration of both UDCA and cholestyramine, along with vitamin K supplementation, allowed for partial symptomatic control and reduction of potential therapy-related complications. Multidisciplinary management, including close maternal and fetal monitoring, timely administration of antenatal corticosteroids, and planned delivery between 32 and 34 weeks, was critical in optimizing maternal and neonatal outcomes. This case highlights the importance of individualized, stepwise pharmacologic management combined with vigilant obstetric care in severe or UDCA-resistant ICP.
